# Location, location, location: a discrete choice experiment to inform COVID-19 vaccination programme delivery in the UK

**DOI:** 10.1186/s12889-022-12823-8

**Published:** 2022-03-04

**Authors:** Robert McPhedran, Natalie Gold, Charlotte Bemand, Dale Weston, Rachel Rosen, Robert Scott, Tim Chadborn, Richard Amlôt, Max Mawby, Ben Toombs

**Affiliations:** 1 Kantar Public UK Behavioural Practice, 4 Millbank, Westminster, London, SW1P 3JA UK; 2 Public Health England Behavioural Insights, Public Health England, Wellington House, 133-155 Waterloo Road, London, SE1 8UG UK; 3 Centre for Philosophy of Natural and Social Science, London School of Economics and Political Science, Houghton Street, London, WC2A 2AE UK; 4 Solent NHS Trust, NHS England, Highpoint Venue, Bursledon Rd, Southampton, Hampshire SO19 8BR UK; 5 Behavioural Science and Insights Unit, UK Health Security Agency, Porton Down, Salisbury, Wilts SP4 0JG UK; 6 Economics and Strategic Analysis Team, NHS England and NHS Improvement, Wellington House, 133-155 Waterloo Road, London, SE1 8UG UK

**Keywords:** Covid-19, Discrete choice experiment, SMS, Text message, Vaccination uptake, Young people

## Abstract

**Background:**

Large-scale vaccination is fundamental to combatting COVID-19. In March 2021, the UK’s vaccination programme had delivered vaccines to large proportions of older and more vulnerable population groups; however, there was concern that uptake would be lower among young people. This research was designed to elicit the preferences of 18–29-year-olds regarding key delivery characteristics and assess the influence of these on intentions to get vaccinated, to inform planning for this cohort.

**Methods:**

From 25 March to 2 April 2021, an online sample of 2012 UK adults aged 18–29 years participated in a Discrete Choice Experiment. Participants made six choices, each involving two SMS invitations to book a vaccination appointment and an opt-out. Invitations had four attributes (1 × 5 levels, 3 × 3 levels): delivery mode, appointment timing, proximity, and sender. These were systematically varied according to a d-optimal design. Responses were analysed using a mixed logit model.

**Results:**

The main effects logit model revealed a large alternative-specific constant (β = 1.385, SE = 0.067, *p* < 0.001), indicating a strong preference for ‘opting in’ to appointment invitations. Pharmacies were dispreferred to the local vaccination centre (β = − 0.256, SE = 0.072, *p* < 0.001), appointments in locations that were 30–45 min travel time from one’s premises were dispreferred to locations that were less than 15 min away (β = − 0.408, SE = 0.054, *p* < 0.001), and, compared to invitations from the NHS, SMSs forwarded by ‘a friend’ were dispreferred (β = − 0.615, SE = 0.056, *p* < 0.001) but invitations from the General Practitioner were preferred (β = 0.105, SE = 0.048, *p* = 0.028).

**Conclusions:**

The results indicated that the existing configuration of the UK’s vaccination programme was well-placed to deliver vaccines to 18–29-year-olds; however, some adjustments might enhance acceptance. Local pharmacies were not preferred; long travel times were a disincentive but close proximity (0–15 min from one’s premises) was not necessary; and either the ‘NHS’ or ‘Your GP’ would serve as adequate invitation sources. This research informed COVID-19 policy in the UK, and contributes to a wider body of Discrete Choice Experiment evidence on citizens’ preferences, requirements and predicted behaviours regarding COVID-19.

**Supplementary Information:**

The online version contains supplementary material available at 10.1186/s12889-022-12823-8.

## Background

### Introduction

Since the detection of COVID-19 in late 2019, mass vaccination programmes have been considered a fundamental component in many nations’ pandemic response strategies. Achieving large-scale, population-level vaccination is critical to combatting the virus, and to ameliorating the extreme health, economic and social impacts that COVID-19 continues to impose. To be effective, such programmes require high uptake.

Governments around the world are at different stages in planning and implementing their vaccination programmes; and at this stage, the availability of vaccines varies considerably. However, authorities in all countries will ultimately need to formulate strategies that maximise vaccines’ accessibility for all population sub-groups and address the reasons for hesitancy [1]. The urgency of this requirement is underscored by the recent emergence of novel variants of COVID-19 with greater transmissibility, which have the potential to derail pandemic exit strategies and burden health systems [2]; and by the risk of future variants which may prove resistant to current vaccines [3].

The United Kingdom (UK) is currently in a fortunate position. The Government’s COVID-19 mass vaccination programme – which began on 8th December 2020 – has been amongst the most successful globally in delivering the availability, accessibility and motivation required. At the time of writing, more than 75 million doses of three of the programme’s approved vaccines – Pfizer-BioNTech, Oxford-AstraZeneca and Moderna – have been administered across the UK, with exceptionally high acceptance rates observed (Gov.uk, 2021). However, despite this unequivocally positive progress, concerns had been raised that there was likely to be a decrease in uptake once the programme progresses to population groups who may be less likely to accept vaccines. One of these groups is younger people [4]: according to recent research from the UK’s Office for National Statistics (ONS), intent to receive a COVID-19 vaccine is lowest among those aged 16–29 years [5]. Further, these results have been corroborated by other large-scale UK household surveys [6].

The reasons for lower levels of intent among younger people are numerous and varied; however, the ways in which vaccines are made available to younger people are likely to be an important determinant of levels of uptake. According to research conducted by the UK’s Royal Society of Public Health (RSPH), inconvenient scheduling of appointments and delivery location serve as barriers to lifetime vaccine uptake [7]. More specifically, research into COVID-19 vaccination has highlighted that programme characteristics such as appointment scheduling and administration location are important in facilitating uptake, both in the UK [8, 9] and elsewhere [10].

Given this, a comprehensive, multi-faceted, evidence-based delivery strategy which accounts for the unique preferences of 18–29-year-olds is required to ensure sufficient levels of uptake among this age group. Previous research on the attitudes and responses of young people towards vaccines and vaccination programmes exists (e.g. [11]). However, COVID-19 is a singular virus which has impacted lives on a different scale, and new types of vaccines have been needed. Likewise, the delivery of the UK’s vaccination programme to date has provided substantial data and experience from which to draw insights; but it has largely focussed on older age groups and people presenting higher risk factors. For both these reasons, it was felt that knowledge and experience relating to other pathogens and vaccination programmes, and different population groups, could not alone confidently be applied to the delivery strategy for young people.

Therefore, a multi-disciplinary team – comprising members from Kantar Public UK’s Behavioural Practice, Public Health England (PHE), NHS England (NHSE) and the Department of Health and Social Care (DHSC) – was assembled to design and implement research to ensure that the strategy was predicated on a robust evidence base. A discrete choice experiment (DCE) was then chosen as the method to elicit the preferences of 18–29-year-olds regarding key delivery characteristics, and to assess the influence of these characteristics on intentions to get vaccinated, in order to inform decisions about specific elements of a delivery strategy that would maximise take-up across this age group.

### Discrete choice experiments

DCEs present participants with a series of choices between options that differ in terms of chosen attributes. Based on participants’ selection of options, preferences with respect to attributes are extracted using a technique based on Thurstone’s Random Utility Theory (RUT), which was extended by McFadden in the late twentieth century [12]. One of RUT’s core assumptions is that choice is underpinned by a ‘utility’, which has systematic and random components. Systematic components comprise latent values attached to attributes (and levels of each attribute) in choice alternatives, as well as covariates that are influential in selection. In contrast, random components comprise unknown factors that may impact decisions [13].

DCEs have been increasingly applied in public health research to understand citizens’ preferences with respect to interventions or programmes [14], and empirical research has demonstrated their robust external validity: for example, they have been used to accurately predict medical treatment [15] and vaccination behaviour [16]. During the pandemic, DCEs have been used to understand which vaccine characteristics are most influential in decision-making [17]; health and economic trade-offs in lockdowns [18]; and exit strategies [19].

Given their application across domains in public health research and strong external validity, a DCE was an appropriate method to understand the preferences of 18–29-year-olds with respect to key delivery characteristics, to provide evidence for which aspects of existing services might need to be adjusted and which new options might be required.

## Methods

### Discrete choice experiment attributes, experimental design and operationalisation

In contrast to many other public health DCEs – which often rely on primary qualitative research to inform design (see [14]) – this study’s DCE was envisaged and designed in collaboration with individuals responsible for the strategic planning of the UK’s mass vaccination programme. The design of the DCE was informed by three sessions in the weeks commencing 8th and 15th March 2021; representatives from Kantar Public UK, PHE, NHSE and DHSC were present at each session.

The DCE presented participants with a series of appointment scenarios, each comprising varying combinations of levels under the four attributes described below. The scenarios were shown in pairs, in the format of an invitation to book an appointment to receive a vaccine. Participants were told: ‘In each pair, we’d like you to select the text message that is most likely to prompt you to log in and book an appointment. You can also select ‘neither appointment’ if you don’t wish to receive a coronavirus vaccine or if neither of the options are acceptable to you.’ Choosing to book an appointment (by virtue of rejecting the option to select neither) was used as an immediate proxy for vaccine take-up, as it was presented to participants as the first in a chain of actions that led to getting a vaccination.

The final design comprised four attributes (1 × 5 levels, 3 × 3 levels), each of which can be seen in Table 1. The rationale for the definition of attributes and levels was as follows:

**Table 1:** DCE attributes and levels

Attribute	Level 1	Level 2	Level 3	Level 4	Level 5
**Mode of delivery**	Local vaccination centre	Nearby GP surgery (Primary Care Network)	Nearby pharmacy	Drive-thru	Mobile/ pop-up
**Appointment time**	Monday to Friday, 9 am-5 pm	Monday to Friday, after hours (before 9 am or after 5 pm)	Weekends		
**Proximity from one’s home**	Less than 15 min	Between 15 and 30 min	Between 30 and 45 min		
**SMS invitation sender**	NHS	Your GP	Best friend		

The five attributes (and their levels) included in the DCE design

#### Mode of delivery

Mechanisms for vaccination through Primary Care Networks (PCNs) and Local Vaccination Centres were already well established as part of the first phase of the programme, and it was assumed that these would continue to play an important role in providing access for young people. There was also recognition of the likely need for more targeted, localised services: recruitment of local pharmacies had recently begun, and there was interest in the potential utility of mobile services (which could be deployed in convenient locations) as well as drive-through options. Therefore, the rationale for the inclusion of this attribute was to understand whether these options should be employed – or scaled up or down – according to young people’s preferences.

#### Appointment time

Existing research shows that greater convenience plays a role in reducing hesitancy [20]; but evidence on whether extended opening hours are advantageous for young people specifically is lacking. Additionally, as noted, COVID-19 presents a novel situation in which past experiences might not apply. Services had been commissioned to operate 7 days a week with extended opening hours to heighten convenience. The rationale for the inclusion of this attribute was therefore to identify any strong preferences among young people for appointments outside of normal working hours, further to inform resource planning and allocation.

#### Proximity

Evidence for a distance decay effect, whereby people who live further away from healthcare facilities have lower levels of usage after adjustment for need [21], suggests that more proximate vaccination locations will result in higher levels of uptake. Vaccination Centres had been specifically situated within 45 min of 99% of the population in England. As such, the rationale for the inclusion of this attribute was to identify any impacts of travel times ranging from 0 to 45 min on young people’s propensity to attend a vaccination appointment.

#### SMS invitation sender

Unpublished observations had indicated that SMS text messages from friends and family may be effective in motivating attendance at appointments among younger people; General Practice (GP) text messaging systems were already in operation, and a national SMS booking system was about to go live. Consequently, the rationale for the inclusion of this attribute was to provide evidence of any difference in motivational impact due to these SMS senders, to inform planning.

A full factorial design with these attributes/levels would have included *n* = 135 profiles (5^1^ * 3^3^), the presentation of which would have been infeasible in this study. Therefore, a d-optimal fractional factorial design was generated using the *choiceDes* package in R Statistical Software.

**Fig. 1:**
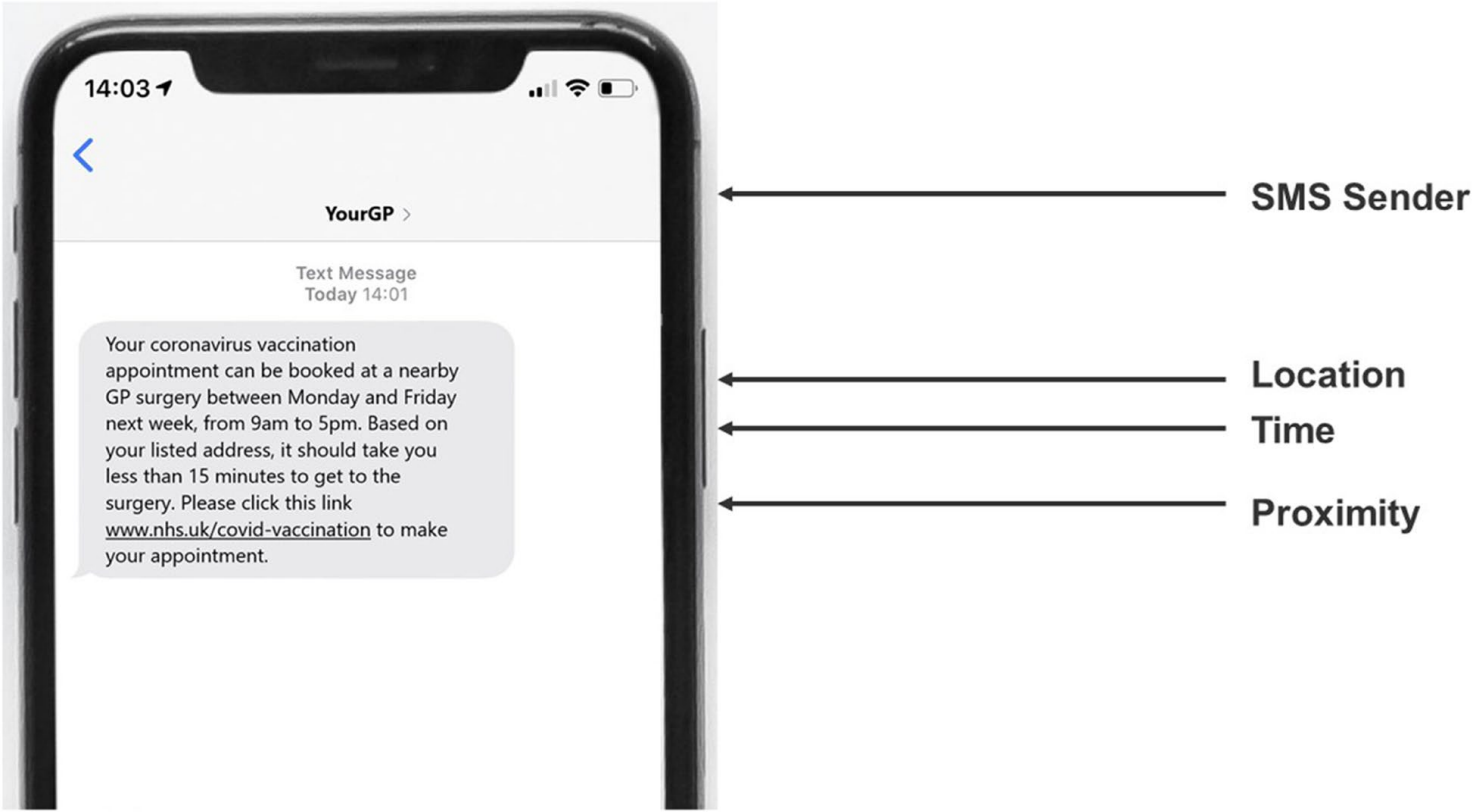
Example SMS message**.** “The DCE comprised six pairs of SMS invitations, the attributes of which differed systematically according to the d-optimal fractional factorial design”

The final design was unlabelled and comprised *n* = 6 paired choice sets, each of which contained an opt-out to maximise external validity. Following the finalisation of the design, each of the choices was translated into an image of an invitation SMS message for presentation in the DCE, an example of which can be seen in Fig. 1. Below. Details describing the mode of delivery, appointment time and proximity attributes were built into the SMS text. SMS invitation sender was identified at the top of the screen. For choices incorporating the ‘best friend’ level, the SMS was presented as having been forwarded on by a friend with a supplementary motivational message, rather than as having originated from the friend. This ‘sender’ was included to test the potential impact of a social norms-based intervention originating from strong social ties [22].

The use of pictorial choice options arguably delivers a more natural, relatable and engaging set of stimuli for participants than the more usual tabular format for DCEs. As Kahneman and Tversky have argued, “the method of hypothetical choices… relies on the assumption that people often know how they would behave in actual situations of choice, and on the further assumption that the subjects have no special reason to disguise their true preferences” [23]. The method used in this study brings participants closer to the “actual situation of choice” than is often the case with DCEs, enhancing its external validity. Further, maximising engagement with the experiment was deemed to be especially important given expectations of lower engagement among the 18–29 age group [24]. However, despite the use of a communications medium to represent vaccination options, the experiment was focussed on informing urgent strategic decisions about vaccine delivery rather than tactical choices about communications approaches.

Before starting the DCE, participants were provided with an overview of the scenario (detailing the vaccination programme) and their choice task, which involved selecting the vaccination appointment that they would be most likely to book based on its characteristics, or selecting ‘neither appointment’.

**Fig. 2:**
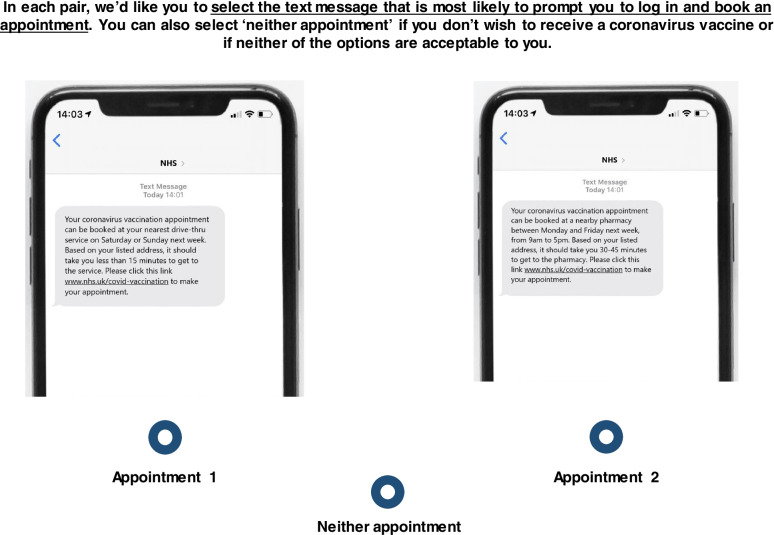
Example paired choice set. **“**Participants completed six paired pictorial choice sets, the order of which was randomised. Each choice set also contained an opt-out: ‘Neither appointment’”

The introduction to the DCE and the complete choice set can be seen in the Additional file 1, and an example of a paired choice set can be seen in Fig. 2.

In the experiment, the order in which the pairs were shown to participants was randomised to minimise the influence of order effects [25].

### Participants

#### Sample size requirement

There is no scientific consensus on the sample size required for a sufficiently powered DCE. However, rules of thumb have been proposed in the literature, the most common of which is that from Johnson and Orme [26]. According to the authors, the sample size required for a main effects DCE model can be calculated using the following equation:

$$N>\frac{500c}{\left(t\ast a\right)}$$

Where: a represents the number of alternatives (2); t represents the number of choice tasks (6), and c represents the number of levels in the largest attribute (5). According to this rule of thumb, we required a minimum of 209 participants, a total which we exceeded in our final sample.

#### Sample

This study was conducted online from 25 March to 2 April 2021, with sample sourced from LifePoints (Kantar’s – an international private sector research company – online access panel).

The sample for the experiment comprised *n* = 2012 adults aged 18–29 years who were living in the UK and had not been vaccinated at the time of interview. To ensure that the sample was nationally representative of this age group in terms of key demographic characteristics, we enforced flexible parallel quotas on age and ethnicity. These quotas were based on mid-year population statistics from the ONS [27]. As an incentive for participation in the study, all participants were provided with LifePoints reward points, which are online tokens that can be redeemed in the form of e-gift cards and PayPal credit.

### Statistical methods

Participants’ choices were analysed using mixed logit models (alternatively termed a random parameters model), adjusted for their panel nature. The model of primary interest was the main effects model, as this represented the preferences of the sub-population. Attributes were set as random parameters – each with a normal distribution – to allow for preference heterogeneity across participants [28]. A likelihood ratio test was conducted to test for a difference in fit between a model which allowed for correlations between random parameters using Choleski decomposition; however, this test did not indicate significant improvement (χ^2^(55) = 31.149, *p* = 0.996), so the non-correlated model was selected for use. This model was estimated in R statistical software using the *mlogit* package [29], and can be written as:

$${\displaystyle \begin{array}{c}U=\alpha +{\beta}_1{w}_1+{\beta}_2{w}_2+{\beta}_3{w}_3+{\beta}_4{w}_4+{\beta}_5{w}_5+{\beta}_6{x}_1+{\beta}_7{x}_2+{\beta}_8{x}_3\\ {}+{\beta}_9{y}_1+{\beta}_{10}{y}_2+{\beta}_{11}{y}_3+{\beta}_{12}{z}_1+{\beta}_{13}{z}_2+{\beta}_{14}{z}_3+\varepsilon \end{array}}$$

Where:

- *α* denotes the model alternative specific constant (ASC; the systematic preference for ‘opting in’ to appointment options).
- *β*_1_ − *β*_5_ denote individual-specific coefficients representing the effect of vaccination delivery modes (w_1_ represents ‘Vaccination centre’, w_2_ represents ‘GP surgery’, w_3_ represents ‘Nearby pharmacy’, w_4_ represents ‘Drive-thru’, w_5_ represents ‘Mobile/pop-up’) on selection.
- *β*_6_ − *β*_8_ denote individual-specific coefficients representing the effect of appointment times (x_1_ represents ‘Monday to Friday, 9am-5pm’; x_2_ represents ‘Monday to Friday, after hours (before 9am or after 5pm)’, x_3_ represents ‘Weekends’) on selection.
- *β*_9_ − *β*_11_ denote individual-specific coefficients representing the effect of venue proximity (y_1_ represents ‘Less than 15m’, y_2_ represents ‘Between 15 and 30m’, y_3_ represents ‘Between 30 and 45m’) on selection.
- *β*_12_ - *β*_14_ denote individual-specific coefficients representing the effect of invitation sources (z_1_ represents ‘NHS’, z_2_ represents ‘YourGP’, z_3_ represents ‘Best friend’) on selection.
- ε is the random error term, representing the non-systematic component in selection.

All attributes were dummy coded, such that 1 represented their presence in each choice card, while 0 represented their absence. Coefficients’ signs reflect whether a level has a positive or a negative effect on utility compared to the reference category; further, their absolute values indicate their relative importance in selection, again compared to the reference category. To facilitate ease of interpretation, coefficients were exponentiated to generate odds ratios.

In addition to the main effects model, three secondary hybrid models – in which age band was interacted with DCE attributes – were run to explore preferences within the cohort. There were no major differences in preferences according to age band; as such, the results of these models are appended in Additional file 2.

## Results

### Achieved sample profile

As mentioned, the sample for this experiment was *n* = 2012 adults aged 18–29 years (living in the UK and unvaccinated at the time of interview). The profile of our achieved sample – compared to quota targets, where relevant – can be seen in Table 2.

**Table 2:** Achieved sample and quota targets

Demographic		Achieved %	Quota %
Age	18–21	32%	30%
	22–25	33%	34%
	26–29	35%	36%
Ethnicity	White	81%	81%
	Asian, Black Mixed/Other	19%	19%
Gender	Male	49%	–
	Female	50%	
	Identify in a different way	1%	
What is the highest level of education you have completed?	No formal qualifications	3%	–
	GCSE / CSE / O-level; AS-Level; A-Level / Scottish Highers	56%	
	Degree; Masters / MBA / MSc; PhD	41%	

Age and ethnicity parallel quota targets, and the sociodemographic profile of the final achieved sample

### Opt-out analysis

Across the total sets, 83% of choices involved the selection of one of the two available appointments, while 17% of all choices involved ‘opting out’ of the two appointments presented.

**Fig. 3:**
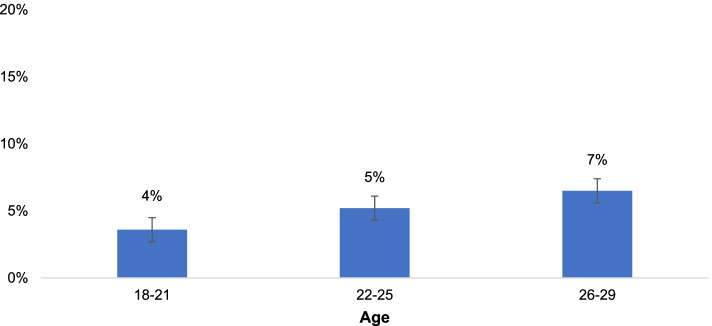
Proportion who opted out in all six paired sets, by age. “The proportion of participants who ‘opted out’ in all six paired choices was similar for each age sub-group: 4% of those aged 18-21 opted out; 5% of those aged 22-25; and 7% of those aged 26-29”

Only 5% of all participants (*n* = 103) opted out in all six paired sets, with similar proportions observed across the three age sub-groups within the sample (see Fig. 3). Differences in opt-out proportions were tested using two-tailed Z-tests for proportions with a Bonferroni-corrected *p*-value; however, no statistically significant differences were observed.

### Mixed logit models

The preference weights for the main effects model are contained in Table 3. In a result consistent with the opt-out figures above, the model’s large ASC coefficient (β = 1.385, SE = 0.067, *p* < 0.001) indicated a strong systematic preference for ‘opting in’ to appointments in the DCE.

**Table 3:** Attribute estimates, random parameters model

Attributes	Levels	Coefficient (β)^**a**^	Standard error	Odds ratio	***P***-value
**Alternative specific constant**		1.385	0.067	3.995	< 0.001
**Delivery mode**	Local vaccination centre	*Reference category*			
	Nearby GP surgery (PCN)	0.105	0.048	1.111	0.028
	Nearby pharmacy	−0.256	0.072	0.774	< 0.001
	Drive-thru	−0.329	0.062	0.720	< 0.001
	Mobile / pop-up	0.003	0.080	1.002	0.973
**Appointment time**	Monday to Friday, 9 am-5 pm	*Reference category*			
	Monday to Friday, after hours	−0.234	0.056	0.791	< 0.001
	Weekends	−0.057	0.038	0.945	0.134
**Proximity from one’s home**	Less than 15 min	*Reference category*			
	Between 15 and 30 min	0.154	0.058	1.166	0.008
	Between 30 and 45 min	−0.408	0.054	0.665	< 0.001
**SMS invitation sender**	NHS	*Reference category*			
	Your GP	−0.065	0.054	0.938	0.232
	Best friend	−0.615	0.056	0.541	< 0.001
**Log-likelihood**	−11,776				
**Akaike Information Criteria**	23,595				

Coefficients, standard errors, odds ratios and *p*-values of the random parameters model

^a^ Represents the mean of all individual-specific coefficients within the total sample (*n* = 2012)

The model also highlighted variation in preferences with respect to appointment characteristics, particularly delivery mode and proximity. In terms of delivery mode, ‘Nearby GP Surgery’ was most preferred (β = 0.105, SE = 0.048, *p* = 0.028), while the difference between ‘Local vaccination centre’ (the reference group) and Mobile/pop-up (β = 0.002, SE = 0.080, *p* = 0.973) was not statistically significant. Conversely, both ‘Nearby pharmacy’ (β = − 0.256, SE = 0.072, *p* < 0.001) and ‘Drive-thru’ (β = − 0.329, SE = 0.062, *p* < 0.001) were less preferred as modes of vaccination. In terms of location proximity, venues located between 15 and 30 min from one’s place of residence (β = 0.154, SE = 0.058, *p* = 0.008) were preferred over those located less than 15 min away. On the other hand, venues located between 30 and 45 min from one’s place of residence were substantially less preferred than closer locations (β = − 0.408, SE = 0.054, *p* < 0.001).

The final two attributes – appointment time and invitation source – were, relatively speaking, less influential in participants’ decision making in the DCE. In terms of the former attribute, appointments scheduled afterhours throughout the week were least preferred (β = − 0.234, SE = 0.056, *p* < 0.001); in terms of the latter, invitations forwarded from one’s best friend were the least favoured (β = − 0.614, SE = 0.056, *p* < 0.001).

There was a generally consistent pattern of preferences in the three hybrid age interaction models; as such, the results of these models are appended in Additional file 2.

### Preference heterogeneity

The distribution of individual-specific coefficients in a mixed logit model provides information about the degree of preference heterogeneity across a given sample.

In this case, direction of preferences – measured in the main effects logit model – was relatively consistent across the sample, but strength of influence often differed (see Table 4). The attribute levels that had the largest positive influence upon appointment selection according to their coefficient – for example, Proximity ‘Between 15 and 30 m’ – positively impacted selection for most in the sample; however, the extent of this impact differed (IQR = 0.257). Similarly, the levels that had the largest negative influence upon appointment selection – for example, SMS invitation sender ‘Best friend’ – had a negative impact for most; however, again, the scale of this varied (IQR = 0.320).

**Table 4:** Range of attribute estimates, random parameters model

		Range of individual-specific coefficients		
Attributes	Levels	1st Quartile	3rd Quartile	Inter-quartile range
**Alternative specific constant**		1.107	1.663	0.556
**Mode of delivery**	Local vaccination centre	*Reference category*		
	Nearby GP surgery (PCN)	0.085	0.126	0.041
	Nearby pharmacy	−0.510	− 0.002	0.508
	Drive-thru	−0.533	−0.125	0.408
	Mobile /pop-up	−0.070	0.076	0.146
**Appointment time**	Monday to Friday, 9 am-5 pm	*Reference category*		
	Monday to Friday, after hours	−0.301	−0.168	0.133
	Weekends	−0.073	−0.041	0.032
**Proximity from one’s home**	Less than 15 min	*Reference category*		
	Between 15 and 30 min	0.026	0.283	0.257
	Between 30 and 45 min	− 0.421	− 0.395	0.026
**SMS invitation sender**	NHS	*Reference category*		
	Your GP	−0.243	0.113	0.356
	Best friend	−0.775	−0.455	0.320

Distribution statistics of individual-specific coefficients: first quartile, third quartile and inter-quartile range

There was, however, one attribute level whose presence polarised respondents more than others: Your GP as an invitation sender. For Your GP, the first quartile was negative (Q_1_ = − 0.243) suggesting a preference for a message from the NHS; on the other hand, the third quartile was positive (Q_3_ = 0.113), suggesting a preference for a message from Your GP. This result suggests that further research is required to determine the appropriate SMS messenger for vaccination invitations.

### External validity

In order to evaluate the external validity of the model, the coverage rate for two plausible scenarios – that is, scenarios close to the approach used within the existing programme – were calculated (see Table 5; for additional details on the calculation of the coverage rate, see Additional file 2). These two scenarios both involved delivery of vaccines at a Vaccination Centre between 9 am and 5 pm on Monday to Friday, with the NHS as the invitation source. The proximity differed: in one scenario the Vaccination Centre was defined as less than 15 min’ from home; in the other it was between 15 and 30 min from home. The predicted coverage rates for these two scenarios were 79 and 81% respectively. Data from NHS England shows that 80% of 18–29-year-olds had received their first vaccine dose by 2 January 2022 [30].

**Table 5:** Predicted coverage rate

Scenarios				Predicted coverage rate
Mode of delivery	Appointment time	Proximity from one’s home	SMS invitation sender	
Local vaccination centre	Monday to Friday, 9 am-5 pm	Less than 15 min	NHS	79%
Local vaccination centre	Monday to Friday, 9 am-5 pm	Between 15 and 30 min	NHS	81%

The predicted coverage rates for two ‘realistic’ scenarios – 79% for the first scenario, and 81% for the second scenario – were similar to recent uptake statistics, thereby providing evidence for the external validity of this study

## Discussion

This study aimed to identify the preferences of 18–29-year-olds with respect to key delivery characteristics in the UK’s vaccination programme. The study used a novel pictorial DCE to understand the relative importance of delivery mode, proximity, timing and invitation source in this sub-population’s consideration of vaccination appointments, thereby helping to inform programme planning and delivery.

Overall, there was a strong systematic preference for ‘opting in’ to the appointment invitations in the DCE, suggesting that most aged 18–29 will choose to accept an appointment when invited given the presented programme characteristics. However, results did highlight preferences with respect to vaccine delivery, particularly in terms of delivery mode. While GP surgeries were the most preferred options for vaccine administration, Vaccination Centres and mobile pop-ups did not serve as disincentives. This result is positive given that delivery mechanisms for the former two location types are already well-established in the current vaccination programme: as of 28th May 2021, in England there were 164 Vaccination Centres and 1027 GP-led services [31].

However, pharmacies – of which there were 524 in the vaccination programme, with further recruitment underway – were not preferred options for vaccine administration. As Table 4 indicates, there was considerable variation in this across the sample (more so, in fact, than for any other delivery characteristic), and while some participants displayed a strong preference for Vaccination Centres over pharmacies, for others the preference was slight.

The overall result is at odds with some of the empirical literature: previous research conducted in England demonstrated that community pharmacies can increase vaccination uptake [32]. However, as noted elsewhere, COVID-19 presents novel situations and challenges, and 18–29 s are a specific population sub-group, so there is no guarantee that evidence from other sources or regarding other sub-groups will apply equally in this context. Moreover, other research conducted in the UK has indicated that individuals may possess lower levels of trust in pharmacists, particularly when they are delivering ‘unfamiliar’ services considered to be high risk [33]. One may speculate that 18–29 s are indeed less familiar with pharmacies than other groups in the population – although further research would be required to ascertain this.

Proximity was another attribute that strongly influenced participants’ choices in the DCE: there was a preference for administration locations less than half an hour from one’s place of residence, particularly those 15–30 min away. The desire for closer locations for medical care is not unusual, as similar DCEs elsewhere have noted [34]. As discussed, Vaccination Centres are located to be within 45 min of 99% of the population, so while Vaccination Centres should be expected to be preferred as modes of delivery in themselves, for some 18–29 s they may be too far away to be viable options.

However, the overall preference for a location 15–30 min away – rather than less than 15 min away – is atypical and counter to expectations. First, it should be noted that this result was not consistent across the sample: as Table 4 indicates, preferences within the first quartile were slight. Any explanation for this result would be speculation, but it may be that many were averse to the idea of large numbers of people coming to a location close to their home to receive a vaccine. Alternatively, it is possible that individuals were thinking of preferred vaccination locations near their actual place of residence, and assumed a travel time of 15–30 min (which is the approximate time it would take for a majority of the population to access vaccination locations: [35]). Further research would therefore be required to provide answers to this question.

The timing of appointments had a less pronounced influence on participants’ choices, but there was a consistent preference for those scheduled during between 9 am and 5 pm on Monday to Friday, and at weekends, as opposed to ‘after hours’ on Monday to Friday. This provides some evidence with which to address the question of whether extended opening hours would be required to encourage 18–29-year-olds to make appointments to receive a vaccine. It suggests that whilst extended hours may be appropriate for certain sub-groups, normal working hours should continue to be a key priority in resource allocation.

Despite indications from unpublished work that SMS text messages from friends may be motivating, the DCE revealed a strong negative reaction to this approach among 18–29-year-olds, with a clear preference for one’s GP and/or the NHS. The DCE did not test SMS text messages as a medium against other possible channels (all choice options were presented in as text messages, while the messenger varied). However, the fact that these choice options collectively generated a strong preference for ‘opting in’ to vaccination suggests that existing GP text messaging systems and the forthcoming national SMS booking system would both be effective channels for communication.

Finally, the congruence between the level of appointment uptake predicted by the DCE model (79 and 81% in the two scenarios) and actual levels of first dose uptake recorded by the end of 2021 (80%) demonstrate the external validity of the model, and provide further evidence for the ability of DCEs to provide accurate predictions of health behaviours (see [15, 16]).

In addition to helping to inform the UK Government’s strategy, this research contributes to the wider body of public health literature in three ways. It is the first study that focuses exclusively on identifying COVID-19 vaccination programme attributes important to those aged 18–29; therefore, it may be useful for public health policymakers and practitioners globally who are formulating COVID-19 vaccination strategies aimed at younger people. Second, the study further demonstrates the value in applying discrete choice experiments to inform health policy in a live, rapidly changing setting. Third, its design involving a novel DCE presentation format, with choices operationalised in pictorial form, may be used in future to optimise a variety of interventions relating to public health programmes including – but not limited to – Short Message Service (SMS) invitations and communications materials.

## Strengths and limitations

Some limitations in this study need to be acknowledged. Perhaps most obviously (given the discussion above), while this research can provide evidence of preferences between delivery characteristics, it cannot reveal the reasons underpinning those preferences. For example, the reasons for the deterrent effect of pharmacies, and of the shortest travel times, need to be inferred or hypothesised. Further research into these questions would be needed if explanations are required.

Second, to enable rapid collection of data and provision of evidence, this study’s sample was drawn from an online access panel. Thus, people without access to the internet were necessarily excluded, and the sample may have been open to selection biases inherent to online panels. However, given that 99.5% of people in the age group in question had used the internet within the past 3 months in 2020 [27], impacts of the exclusion can be discounted; and steps were taken to minimise biases within the sample by setting and meeting quotas for age and ethnic sub-groups.

The study’s strengths should equally be highlighted. First, the DCE was designed in close collaboration with vaccination policy and implementation specialists in Government. The DCE’s attributes and levels were chosen to relate to existing provision and potential new delivery options based on practical capabilities and evidence of what has been effective in other contexts. As such, it was specifically designed to answer live questions about the delivery of COVID-19 vaccines to 18–29-year-olds, and thus to inform decision-making regarding the continued roll-out of the vaccination programme in England. Second, the sample size was large compared with many DCEs relating to healthcare, providing sensitivity to detect small differences in preference. Third, fieldwork was completed within one week, ensuring a comparable context across the sample despite the rapidly evolving environment. Fourth, the use of pictorial choice options (SMS text messages expressing the characteristics of each appointment type) arguably delivers a more engaging, natural and relatable set of stimuli for participants than the more usual tabular format for DCEs, thereby enhancing external validity.

## Conclusions

Read as a whole, the results of this DCE suggest that the current configuration of the UK’s mass vaccination programme is well-placed to deliver vaccines to 18–29-year-olds. Preferences for receiving a vaccine, and for existing delivery modes (GP surgeries and Vaccination Centres) were strong – provided distance from less proximate Vaccination Centres is not a disincentive. This indicates that resources should continue to be deployed in their current form. However, the DCE also provides evidence that suggests answers to some of the specific questions regarding the needs of 18–29-year-olds. In particular, the assumption that convenience is a key driver of uptake needs to be examined further.

## Supplementary Information

**Additional file 1.**

**Additional file 2.**

## Data Availability

The full datasets generated and/or analysed during the current study are not publicly available, as the research was conducted on behalf of the UK Government. However, a reduced dataset is available from the corresponding author on reasonable request.

## Abbreviations

ASC: Alternative Specific Constant
DHSC: Department of Health and Social Care
DCE: Discrete Choice Experiment
GP: General Practitioner
NHS: National Health Service
NHSE: NHS England
ONS: Office for National Statistics
PCNs: Primary Care Networks
PHE: Public Health England
RUT: Random Utility Theory
SMS: Short Messaging Service
UK: United Kingdom
VCs: Vaccination Centres
