# Supplementary material for: Location, location, location: a discrete choice experiment to inform COVID-19 vaccination programme delivery in the UK
Source: BMC Public Health. 2022 Mar 4;22:431. doi: 10.1186/s12889-022-12823-8 (PMC8894545; doi:10.1186/s12889-022-12823-8)
Supplement: Supplementary file 1 — Additional file 1. [file 12889_2022_12823_MOESM1_ESM.docx]

| **Intro**   \| In the next section of this survey, we’d like you to imagine that it is early summer, **around two months from now**.  National coronavirus restrictions are easing, and people are now allowed to return to work, universities and schools. You can meet with your family and friends outside in parks and gardens. Shops have re-opened, and service at pubs and restaurants has resumed in outdoor areas.  Over the past few months, your older relatives (aged 50+) have all been invited to schedule an appointment to receive a coronavirus vaccine, and now it is your turn. \| \| --- \| |
| --- | --- |
| On the following **six screens** are pairs of text messages, each of which gives information about available appointment slots and provides you with a link to log in and book your appointment.  In each pair, we’d like you to **select the text message that is most likely to prompt you to log in and book an appointment**. You can also select ‘neither appointment’ if you don’t wish to receive a coronavirus vaccine or if neither of the options are acceptable to you.  Ready? Let’s go!  **Design**   \| **** \|  \| \|  \|  \|  \|  \| \| --- \| --- \| --- \| --- \| --- \| --- \| --- \| \|  \|  \|  \| \|  \|  \|  \| \|  \|  \|  \| \|  \|  \|  \| \|  \|  \|  \| \|  \|  \|  \| \|  \|  \|  \| \|  \|  \|  \| \|  \|  \|  \| \|  \|  \|  \| \|  \|  \|  \| \|  \|  \|  \| \|  \|  \|  \| \|  \|  \|  \| \|  \|  \|  \| \|  \|  \|  \| \|  \|  \|  \| \|  \|  \|  \| \|  \|  \|  \| \|  \|  \|  \| \|  \|  \|  \| \|  \|  \|  \| \|  \|  \|  \| \|  \|  \|  \| |
