# Supplementary material for: Location, location, location: a discrete choice experiment to inform COVID-19 vaccination programme delivery in the UK
Source: BMC Public Health. 2022 Mar 4;22:431. doi: 10.1186/s12889-022-12823-8 (PMC8894545; doi:10.1186/s12889-022-12823-8)
Supplement: Supplementary file 2 — Additional file 2. [file 12889_2022_12823_MOESM2_ESM.docx]

**Mixed logit age band interaction models**

**Secondary model 1: 18-21 interaction model**

| **Attributes** | **Levels** | **Coefficient (β)*** | **Standard error** | **Odds ratio** | **P-value** |
| --- | --- | --- | --- | --- | --- |
| **Alternative specific constant***18-21 | | 1.455 | 0.130 | 4.282 | <0.001 |
| **Delivery mode** | Local vaccination centre | *Reference category* | | | |
|  | Nearby GP surgery (PCN) *18-21 | 0.039 | 0.098 | 1.040 | 0.692 |
|  | Nearby pharmacy*18-21 | -0.232 | 0.128 | 0.793 | 0.070 |
|  | Drive-thru*18-21 | -0.214 | 0.111 | 0.807 | 0.054 |
|  | Mobile /  pop-up*18-21 | 0.074 | 0.150 | 1.077 | 0.622 |
| **Appointment time** | Monday to Friday, 9am-5pm | *Reference category* | | | |
|  | Monday to Friday, after hours*18-21 | -0.211 | 0.106 | 0.810 | 0.047 |
|  | Weekends*18-21 | -0.119 | 0.082 | 0.888 | 0.146 |
| **Proximity from one’s home** | Less than 15 minutes | *Reference category* | | | |
|  | Between 15 and 30 minutes*18-21 | 0.101 | 0.113 | 1.106 | 0.371 |
|  | Between 30 and 45 minutes*18-21 | -0.470 | 0.116 | 0.625 | <0.001 |
| **SMS invitation sender** | NHS | *Reference category* | | | |
|  | Your GP*18-21 | -0.186 | 0.112 | 0.830 | 0.098 |
|  | Best friend*18-21 | -0.758 | 0.110 | 0.468 | <0.001 |
| **Log-likelihood** | -3782 | | | | |
| **Akaike Information Criteria** | 7608 | | | | |

**Secondary model 2: 22-25 interaction model**

| **Attributes** | **Levels** | **Coefficient (β)*** | **Standard error** | **Odds ratio** | **P-value** |
| --- | --- | --- | --- | --- | --- |
| **Alternative specific constant***22-25 | | 1.376 | 0.113 | 3.958 | <0.001 |
| **Delivery mode** | Local vaccination centre | *Reference category* | | | |
|  | Nearby GP surgery (PCN) *22-25 | 0.166 | 0.092 | 1.181 | 0.072 |
|  | Nearby pharmacy*22-25 | -0.305 | 0.142 | 0.737 | 0.032 |
|  | Drive-thru*22-25 | -0.314 | 0.113 | 0.731 | 0.006 |
|  | Mobile /  pop-up*22-25 | 0.095 | 0.158 | 1.099 | 0.550 |
| **Appointment time** | Monday to Friday, 9am-5pm | *Reference category* | | | |
|  | Monday to Friday, after hours*22-25 | -0.229 | 0.110 | 0.795 | 0.037 |
|  | Weekends*22-25 | -0.104 | 0.075 | 0.901 | 0.166 |
| **Proximity from one’s home** | Less than 15 minutes | *Reference category* | | | |
|  | Between 15 and 30 minutes*22-25 | 0.216 | 0.131 | 1.241 | 0.100 |
|  | Between 30 and 45 minutes*22-25 | -0.335 | 0.100 | 0.715 | <0.001 |
| **SMS invitation sender** | NHS | *Reference category* | | | |
|  | Your GP*22-25 | -0.082 | 0.103 | 0.921 | 0.427 |
|  | Best friend*22-25 | -0.605 | 0.109 | 0.546 | <0.001 |
| **Log-likelihood** | -3831 | | | | |
| **Akaike Information Criteria** | 7706 | | | | |

**Secondary model 3: 26-29 interaction model**

| **Attributes** | **Levels** | **Coefficient (β)*** | **Standard error** | **Odds ratio** | **P-value** |
| --- | --- | --- | --- | --- | --- |
| **Alternative specific constant***26-29 | | 1.264 | 0.095 | 3.541 | <0.001 |
| **Delivery mode** | Local vaccination centre | *Reference category* | | | |
|  | Nearby GP surgery (PCN) *26-29 | 0.118 | 0.080 | 1.125 | 0.142 |
|  | Nearby pharmacy*26-29 | -0.241 | 0.133 | 0.786 | 0.070 |
|  | Drive-thru*26-29 | -0.373 | 0.106 | 0.689 | <0.001 |
|  | Mobile /  pop-up*26-29 | -0.077 | 0.136 | 0.926 | 0.574 |
| **Appointment time** | Monday to Friday, 9am-5pm*26-29 | *Reference category* | | | |
|  | Monday to Friday, after hours*26-29 | -0.284 | 0.093 | 0.753 | 0.002 |
|  | Weekends*26-29 | -0.010 | 0.068 | 0.990 | 0.878 |
| **Proximity from one’s home** | Less than 15 minutes | *Reference category* | | | |
|  | Between 15 and 30 minutes*26-29 | 0.248 | 0.107 | 1.282 | 0.020 |
|  | Between 30 and 45 minutes*26-29 | -0.429 | 0.091 | 0.651 | <0.001 |
| **SMS invitation sender** | NHS | *Reference category* | | | |
|  | Your GP*26-29 | 0.034 | 0.090 | 1.034 | 0.706 |
|  | Best friend*26-29 | -0.574 | 0.090 | 0.563 | <0.001 |
| **Log-likelihood** | -4150 | | | | |
| **Akaike Information Criteria** | 8343 | | | | |

**Calculation of the coverage rate**

As part of the secondary analysis conducted for this study, the projected coverage (uptake) rates of two scenarios were calculated.

The coverage rate associated with a scenario can be calculated using the formula 1/(1+exp^-u^). Given the use of a mixed logit (random parameters) model in analysis, the distribution of coefficients must be incorporated in such calculations. Accordingly, a simulation with n=5,000 draws – drawing on the model’s mean and standard deviation for each coefficient – was used. In each of these n=5,000 draws, scenario utility values and coverage rates were calculated; average coverage rates for each scenario were then derived from these values.
